# Observations upon the Use of Certain New Remedies

**Published:** 1852-12

**Authors:** L. A. Dugas

**Affiliations:** Professor, &c.


### SELECTIONS.

Trans. of the Med. Soc. of the State of Georgia.

*Observations upon the use of certain New Remedies.* By L. A. Dugas, M. D., Professor, &c.

Believing it to be the duty of members of associations of this kind to make known such results of their observation as may be useful, I will beg leave to offer a few remarks upon the use of some of the medical agents recently brought into notice.

*Chloride of Sodium*, or *common salt*, has been, at various periods, proposed as a valuable remedy, but has attracted more than usual attention during the last twelve months.

I have long been in the habit of prescribing it alone, or in combination with the Bi-carbonate of soda, in certain forms of dyspepsia and general debility. From 10 to 30 grains of salt in a tumbler full of cold water, taken every morning on rising from bed, is highly promotive of appetite and digestion, when the dyspepsia is unattended with organic lesion of the gastric surface and seems to be rather dependent upon a state of atony. The soda, in an equal quantity, constitutes an useful addition where there is a tendency to acidity, and also when the kidneys do not secrete freely and properly. The remedy appears to be especially applicable to the cases of general debility and nervous irritability so common among our ladies of sedentary habits. It may be sometimes necessary to commence with a smaller dose than just mentioned; but I have stated that suited to the majority of cases.

In Acute Dysentery, I have found common salt often of striking usefulness, but it must in general be given early in order to realize its value. It is now ten years since I first witnessed its efficacy in this disease. I had been attending a mulatto boy, eight years of age, for four or five days without being able to ameliorate his condition. The onset of his attack was attended with high febrile action, which, however, gradually subsided as he became exhausted by the continuance of the countless, bloody, mucous stools, and painful tenesmus. The rectum rejected anodyne enemata as fast as they could be administered. Nothing I could suggest seemed to bring any relief; and I left him one night, thinking his case hopeless. On the next morning I found him wonderfully relieved, and was congratulating myself with the idea that my last prescription had "done the deed," when the boy's father announced that he had taken the liberty to omit my prescription and to give his son last night, in lieu of it, a cup full of strong fish brine, which afforded so much relief that he had just again repeated it. The stools became watery and less frequent, the tenesmus was entirely relieved and the patient convalesced rapidly.

I have ever since that time resorted to salt and water; when the high febrile excitement contra-indicated the immediate resort to anodyne enemata; and when the administration of these failed to give relief. In the early and high febrile stage, it will often subdue the fever and tenesmus most admirably, and the patient will rapidly recover.

The efficacy of this and perhaps of other saline purgatives in dysentery, probably depends upon its combined depletory and revulsive operation. The depletion is derived from the *small* intestines in the form of serous stools, and this action must also relieve, by revulsion, the morbid condition of the *large* intestines. The case loses the painful and alarming character of dysentery and assumes that of ordinary diarrhœa, which will either gradually subside or yield to vegetable astringents combined with opiates.

In the spring and in autumn, when the causes of periodical or paroxysmal fevers combine with the vicissitudes of temperature to produce diseases, in which the phlegmasiæ and the neuroses are so commingled as to re-act injuriously upon each other, we often find dysentery prevailing to so great an extent to constitute an epidemic. If the febrile excitement be then closely observed, it will often be found to present daily exacerbations, which must be prevented by the use of quinine during the remissions, in order that the remedies directed to the local affection may have time to prove beneficial. If the quinine be withheld each febrile exacerbation will aggravate the intestinal inflammation, and this, in its turn, will make the next paroxysm more alarming—until it be too late to avert the fatal result. The same may be said of the so-called epidemics of pneumonia, which are most prevalent in mild winters and in remittent fever districts.

In cases unattended with fever, I am in the habit of resorting, as soon as the bowels are *thoroughly emptied*, to enemata consisting of a teaspoonful of laudanum, or half a grain morphine suspended in half a gill of thin starch or mucilage, repeated until retained long enough to give complete relief. This plan will be found to succeed in the great majority of such cases. Yet, we occasionally encounter one in which the anodyne will not be retained, and it is then that I resort to the salt and water.

The dose that I administer is a teaspoonful of salt in a cupful of water, to be repeated every three or four hours until the stools pass off freely and without tenesmus. If the tenesmus returns, the salt should be again given, but at longer intervals.

I am aware that the sulphate of soda in similar doses has been highly recommended, and I have sometimes used it, as also the sulphate of magnesia, with decided advantage. Common salt, however, is less disagreeable, more convenient, and, I think, more efficacious.

Common salt has been of late urged in France, especially by M. Piorry, as a valuable substitute for quinine in Intermittent Fever; and if it can be thus used advantageously, the discovery would be one of great importance to those who cannot afford to purchase the more costly article. I have prescribed it in only two cases of Intermittent Fever; in one of which it proved successful, the patient having had but one paroxysm after its use was commenced. In the other, which was complicated with tubercular disease of the lungs and intestines, it increased the diarrhœa so much as to cause its discontinuance after the second dose. In these cases a teaspoonful in a tumbler of cold water was ordered three times a day. It is to be hoped that this application will be fully tested by the Profession.

*Lemon Juice in Rheumatism.*—There is perhaps no disease, for the relief of which more remedies have been and are still being continually suggested than rheumatism. The last in the list is lemon juice, of whose efficacy the British Journals are full. It may be known to some of those present, that I have for many years advocated the theory (first suggested by Prof. J. K. Mitchell, of Philadelphia,) of the spinal origin of rheumatic affections, and consequently the use of counter-irritations in the vicinity of the origin of the nerves leading to the seat of pain. My views upon this subject were published in the Southern Medical and Surgical Journal in 1837, and I have seen no reason to change them since. We, however, not unfrequently meet with eases of acute Rheumatism of great intensity, in which the spinal treatment cannot be energetically carried out on account of the inability of the patient to rest in any other position than upon his back. In these cases it is difficult to cup the spine often, and very painful to apply blisters to this region. We are then compelled to resort to other means. I should also add that the spinal treatment is by no means so speedily effectual when large joints are much affected with acute inflammation, as in cases of less violence. This is of itself an additional reason for the use of internal medicine, than which I have found nothing more useful than repeated emetics of tartarized antimony, followed in the evening by full doses of opium or morphine.

Within the last few months, however, I have been induced to try the Lemon Juice in a number of cases of acute rheumatism and in the exacerbations of the chronic form of the disease, and always with most decided advantage. The remedy is very grateful to the palate, and the patients own that they feel better as soon as they begin its use, and worse when omitted. I usually order a tablespoonful of the lemon juice of the shops to be taken every four, three, or two hours, according to the violence of the case and the toleration with which it is received by the stomach and bowels. It seems to promote the action of the kidneys, to keep the bowels solvent, to lessen general excitement, and to diminish pain.

So simple and pleasant a remedy in so formidable an affection is well worthy of farther and systematic trial.

*Collodion* and its kindred preparations, the solution of *Gutta Percha* in *Chloroform* and *Gum Shellac* in *Alcohol*, are agents which promise to be useful. Collodion has been much lauded a an application to Erysipelas, and it is, therefore, proper to hear the testimony against it as well as that adduced in favor of its efficacy. Having recently had charge of a case of this disease, I applied the collodion very early to the part affected and a little beyond the inflamed surface. But the disease extended rapidly from the ear to the face and scalp, and in a few days invaded the entire head and a portion of the neck. The collodion was persevered in to the last, without appearing to exercise any controlling influence whatever. My patient recovered, it is true, but I cannot attribute this result to the local application. This is the only case of erysipelas in which I have used it.

*Solution of Gutta Percha in Chloroform.*—This is made by dropping into a vial containing chloroform small fragments of pure gutta percha until the solution acquires the consistence of thick mucilage. It is then applied with a camel hair pencil, which should afterwards be repeatedly dipped in pure chloroform and carefully wiped with paper or old linen so as to prevent its becoming stiff and unfit for farther use.

I will now relate the result of its application in two cases of cancerous affection.

Mr. L. had been troubled with an epithelial cancer of the lower lip which has resisted all applications for eighteen months. There existed, upon the right side of the median line and at the junction of the skin and mucous surface, a small and thin scale or scab, which would occasionally fall or be rubbed off, leaving a raw surface of exquisite sensibility exposed to irritation until another scab would be formed. Beneath this surface there was an induration about the size of a common pea, or rather a little larger, in which the patient frequently felt a very annoying sense of burning, and sometimes darting pain.

At this stage of the case, as the patient was averse to the knife, he was advised to try the application of collodion, which he diligently persevered in for about six months, applying it three or four times daily. This arrested the farther growth of the disease, relieved its itching and burning, protected its surface from ordinary irritants, but did not heal the denuded surface. He then substituted the solution of gutta percha in chloroform in lieu of the collodion. In a letter to me he thus described its effects:—"In a few days I saw and felt a change in the color of the sore and in the irritation; in a week or ten days, the lump disappeared and the irritation subsided, and in three weeks it was almost entirely healed over; in less than a month it was well, leaving an indentation on the lip."

In a note dated the 1st of this month, (April,) my patient writes me: "I begin to feel a return of it in the same place the cure was made eighteen months ago. Recently a lump has appeared in the lip; it is *hard* and sometimes a little sore—it gives me no trouble yet, but I am afraid of it." I will advise him to use the gutta percha and chloroform again.

The next case was that of Maria, a negress, about 50 years of age, who was sent to me from the country on the 3d of November last, with a cancerous ulceration of the mamma of several months' standing. Both mammary glands were very much atrophied, but the affected one was the smaller of the two, presenting nothing but a mass of scirrhous induration which seemed adherent to the thoracic walls, and in the depressed centre of which the remains of a nipple were to be seen drawn back and ulcerated. The ulcer covered a surface equal to the areola. The axillary glands were much enlarged, and the patient a prey to continual pain, especially at night, which deprived her of sleep.

Feeling satisfied that the knife promised no relief under such circumstances, yet unwilling to send her off without trying something, I put her upon the use of the gutta percha and chloroform, thoroughly coating the whole breast daily with it. The discharge from the ulcer would at first cause the pellicle of gutta percha to become loosened in twenty-four hours, so that the surface had to be cleansed before the application of the remedy. The suppuration, however, gradually lessened until the coating would remain a week—the painting still being made each morning. Under this treatment, the patient was gradually relieved of all pain about the breast and even in the axilla. She slept quietly at night, enjoyed her meals and felt quite well. Her general health improved, and she left at the end of one month, with instructions to continue the treatment perseveringly, and to get her master to inform me of the result. I have had no report from her since, but have learned incidentally that she never applied the remedy after she left here, and placed herself under the charge of some one who professed to be able to cure cancers—with what result I know not.

These two cases are narrated with the simple purpose of directing attention to an application which may stay, if it does not cure, so formidable an affection as cancer.

*Solution of Shellac.*—The costliness of the solutions of gun cotton and of gutta percha renders it desirable to have a cheaper article, that may be used as a substitute in cases which require the consumption of a large quantity of such plastic materials. A solution of shellac in alcohol has therefore been proposed for this purpose. This may be prepared by adding successively small bits of shellac to the alcohol of commerce until enough be dissolved to make a mucilaginous solution.

Some of the French practitioners having attributed to collodion extraordinary antiphlogistic properties when applied over affected joints and other inflammatory affections, even more deeply seated, I determined to try, during last winter, the shellac solution in an old case of Rheumatism, in which most of the joints of the extremities were being successively invaded. The toes, ankles, knees, fingers, wrists and elbows were nearly all alternately implicated—becoming very painful and rapidly swelling, so as to be almost double in size in a day or two. I furnished the patient a bottle of the shellac solution, and ordered it to be painted over and around the joint as soon as it would commence to be painful, and to repeat the application several times a day until a thick coating remained, after which it might be applied only once a day. Under this treatment I was gratified to find that the patient could, in a few hours, arrest the pain and prevent the swelling of the joints to which he made the application. He stated that he never had any thing to give him such prompt and effectual relief, although he had been suffering such attacks every winter for the last ten years.—One joint or another continued to annoy him for a month, during all of which time he resorted to the shellac with the same success.

This is the only case in which I have tried this solution.

